# Quantitative Genetic Analysis of Retinal Degeneration in the Blind Cavefish *Astyanax mexicanus*

**DOI:** 10.1371/journal.pone.0057281

**Published:** 2013-02-20

**Authors:** Kelly E. O'Quin, Masato Yoshizawa, Pooja Doshi, William R. Jeffery

**Affiliations:** Department of Biology, University of Maryland, College Park, Maryland, United States of America; University of Florida, United States of America

## Abstract

The retina is the light-sensitive tissue of the eye that facilitates vision. Mutations within genes affecting eye development and retinal function cause a host of degenerative visual diseases, including retinitis pigmentosa and anophthalmia/microphthalmia. The characin fish *Astyanax mexicanus* includes both eyed (surface fish) and eyeless (cavefish) morphs that initially develop eyes with normal retina; however, early in development, the eyes of cavefish degenerate. Since both surface and cave morphs are members of the same species, they serve as excellent evolutionary mutant models with which to identify genes causing retinal degeneration. In this study, we crossed the eyed and eyeless forms of *A. mexicanus* and quantified the thickness of individual retinal layers among 115 F_2_ hybrid progeny. We used next generation sequencing (RAD-seq) and microsatellite mapping to construct a dense genetic map of the *Astyanax* genome, scan for quantitative trait loci (QTL) affecting retinal thickness, and identify candidate genes within these QTL regions. The map we constructed for *Astyanax* includes nearly 700 markers assembled into 25 linkage groups. Based on our scans with this map, we identified four QTL, one each associated with the thickness of the ganglion, inner nuclear, outer plexiform, and outer nuclear layers of the retina. For all but one QTL, cavefish alleles resulted in a clear reduction in the thickness of the affected layer. Comparative mapping of genetic markers within each QTL revealed that each QTL corresponds to an approximately 35 Mb region of the zebrafish genome. Within each region, we identified several candidate genes associated with the function of each affected retinal layer. Our study is the first to examine *Astyanax* retinal degeneration in the context of QTL mapping. The regions we identify serve as a starting point for future studies on the genetics of retinal degeneration and eye disease using the evolutionary mutant model *Astyanax*.

## Introduction

**Figure 1:**
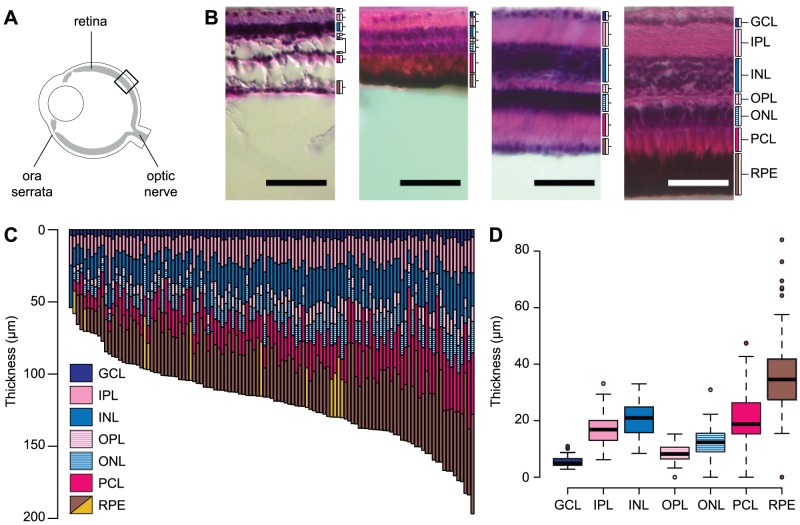
Variation in retinal thickness among *Astyanax* F_2_ hybrids. (A) Diagram of the eye, retina, and retinal landmarks. Black box indicates an example region where we recorded retinal thickness. (B) Example retinal sections from the eyes of four F_2_ hybrids. Scale bar denotes 50 µm and brackets denote individual retinal layers. Cavefish-like retinas (first two panels) were generally thin and may be missing some layers entirely, including the photoreceptor cell layer. Surface fish-like retinas (last two panels) were generally thicker with layers that are well-differentiated and clearly laminated. In some individuals, the retinal pigment epithelium (RPE) was hypopigmented (first and third panels). (C) Retinal thickness among 115 *Astyanax* SFxCF F_2_ hybrids. Yellow bars denote RPEs that were hypopigmented. (D) Box-plots illustrate variance in the thickness of individual retinal layers. The retinal layers shown are: ganglion cell layer (GCL), inner plexiform layer (IPL), inner nuclear layer (INL), outer plexiform layer (OPL), outer nuclear layer (ONL), photoreceptor cell layer (PCL), and retinal pigment epithelium (RPE).

The retina is a thin tissue lining the back of the eye that is responsible for light detection and vision [1]. In vertebrates, retinal precursor cells derived from the neuroectoderm differentiate into six neuronal cell types arranged within seven clearly defined layers. Proceeding from the inside to the outside of the eye, these layers include the ganglion cell layer (GCL), the inner plexiform and nuclear layers (IPL and INL), the outer plexiform and nuclear layers (OPL and ONL), the photoreceptor cell layer (PCL), and the retinal pigment epithelium (RPE) (Figure 1). The PCL contains the outer segments of the photoreceptors, the primary light-sensitive neurons of the retina; the ONL and OPL contain the nuclei of the photoreceptors and their synaptic connections; the INL and IPL contain the amacrine, bipopolar, and horizontal neurons that sum and contrast signals from the photoreceptors, as well as their synaptic connections; and the GCL contains the ganglion cells that transmit the visual signal to the optic nerve. A final layer, the retinal pigment epithelium (RPE), contains no neuronal cells but promotes the development and maintenance of the PCL and reduces glare by absorbing excess light [1]. The neurogenesis of these layers is controlled by a host of genetic and cellular factors (for a review, see [2]). Mutations affecting these and other genes are collectively responsible for many cases of retinal degeneration and blindness in humans (e.g., [3]). Although the most dramatic mutations affecting retinal degeneration have been identified within laboratory model species like mice and zebrafish, evolutionary mutant models–outbred species which exhibit phenotypes that mimic human diseases–can also help dissect the genetic basis of human retinal disease and provide candidates for gene therapy [4].

The Mexican tetra, *Astyanax mexicanus*, is a characin fish distributed throughout the rivers and caves of Northeastern Mexico [5]. The river- or surface-dwelling form of *Astyanax* (surface fish) is typical of most other teleosts, including zebrafish. Surface fish possess large eyes that include a retina which is thick, well-differentiated, and clearly laminated. In contrast, the cave-dwelling form of *Astyanax* (cavefish) exhibits many degenerative traits, including small but nonfunctional eyes buried deep within their orbits [5], [6]. Eye loss has evolved at least twice among 30 populations of *Astyanax* cavefish. Both within and between these populations, cavefish vary in eye size and retinal ultrastructure (for reviews, see [5], [6], [7]). In the least degenerate examples, the cavefish retina retains the GCL, IPL, and INL layers, although the RPE is flattened and the ONL and PCL remain undifferentiated; in the most degenerate examples, the cavefish retina is reduced to a thin strip of undifferentiated tissue or is missing entirely [7], [8]. Because both *Astyanax* surface fish and cavefish are members of the same species, they can be compared to each other in the same way that mutant model species are compared to wild-types in order to identify genes for retinal degeneration [6]. Indeed, this strategy has already proved successful for mapping the mutations responsible for albinism in *Astyanax* [9]. Genetic mapping using surface fish and cavefish hybrids isolated the albino locus to the gene *oculocutaneous albinism type 2* (*oca2*)–the same gene mutated in most cases of human albinism [10].

Although *Astyanax* surface fish and cavefish differ dramatically in the structure of their adult retinas, the early stages of retinal development are surprisingly similar in both morphs. Within the first 70 hours following fertilization, both surface fish and cavefish form all retinal cell types and layers necessary to form the adult retina. Additionally, the retinal progenitor cells of both morphs migrate to the developing retina at the same rate [11], [12]. However, in cavefish, the process of differentiation is generally slower, especially within the PCL [13]; the ventral retina is reduced in size [11]; and cellular differentiation is accompanied by apoptosis in all layers [12]. Beyond 70 hours post fertilization, the synapses of the OPL and IPL form while retinal differentiation and growth continue. But in cavefish, this process, too, is accompanied by a variable degree of apoptosis, especially within the PCL, ONL, and INL [7], [13], [14]. This latter stage of apoptosis produces the wide variability in eye size and retinal ultrastructure observed among adult cavefish. Since *Astyanax* cavefish exhibit reduced eyes and degenerate retina, they serve as an evolutionary mutant model for human ocular diseases such as anopthalmia/microphthalmia and retinitis pigmentosa. Anopthalmia/microphthalmia is a rare genetic disorder characterized by the reduction or loss of eyes [15], while retinitis pigmentosa is a more common genetic disorder characterized by photoreceptor cell death [3]. Although mutations within numerous genes can cause anopthalmia/microphthalmia and retinitis pigmentosa [16], the mutations responsible for many cases remain unknown, including those responsible for up to 30% of all cases of retinitis pigmentosa [3]. Studies using *Astyanax* surface fish and cavefish can help identify these additional loci while also serving as a tractable model to elucidate their etiology [4].

Previous quantitative genetic studies have established that eye degeneration in *Astyanax* is a complex trait caused by numerous mutations of small effect. Quantitative trait loci (QTL) mapping has identified 8–12 QTL for eye size [8], [17], [18]. Presumably, the mutations within these QTL affect developmental ‘eye genes', although mutations within downstream loci such as the opsins are probably involved as well (e.g., [19]). Consistent with these findings, candidate gene studies have revealed that *Astyanax* eye reduction is dependent on early sonic hedgehog expression (*shh*), and that the expression of developmentally important genes such as paired-box proteins (*pax6* and *pax2*) and *ventral anterior homeobox 1* (*vax1*) is modified between surface fish and cavefish [11], [14], [20]; surprisingly, however, none of these genes occur within the QTL so far identified [21]. Other genes involved in retinal function are differentially regulated between surface fish and cavefish, including *LIM homeobox 2* (*lhx2*), *gamma M2c crystallin* (*crygM2c*), and *rhodopsin* (*rho*) (see [6] for a review), but their linkage to cavefish retinal degeneration is unclear. Complicating this scenario, the cavefish lens also undergoes apoptosis, and many cavefish RPE lack melanin. Since both the lens and RPE contribute to the induction and survival of the retina [13], [22], [23], [24], any QTL for eye size presumably includes loci for these confounding factors. For example, RPE hypopigmentation is frequently associated with a suite of retinal maladies, including reduction of rod photoreceptors, thinning in the INL and ONL, and underdevelopment of the GCL [25]. As a consequence of this genetic and regulatory complexity, no previous study has isolated loci specific to retinal degeneration in *Astyanax*.

In this analysis, we use a forward genetic approach coupled with next generation sequencing to identify regions of the *Astyanax* genome responsible for retinal degeneration. We built a genetic map of the *Astyanax* genome using markers developed from sequenced restriction-site associated DNA tags (RAD-seq). We then quantified the thickness of each retinal layer in a group of surface fish x cavefish F_2_ hybrids and searched the genome for QTL associated with retinal degeneration. Finally, we constructed a comparative map of genetic markers between *Astyanax* and zebrafish, which have the same number of haploid chromosomes (n = 25) and share large regions of genomic synteny [26]. We used this comparative map to identify candidate genes for retinal degeneration within each QTL. Our results identified at least four genomic regions responsible for retinal degeneration in *Astyanax* and helped narrow a list of candidate genes specific to the affected retinal layers.

## Materials and Methods

### Genetic Cross and Sampling

We crossed *Astyanax mexicanus* surface fish (surface fish) and cavefish (cavefish) in order to generate an F_2_ hybrid intercross family for quantitative trait loci (QTL) mapping. Yoshizawa et al. [27] describe this cross in detail, but we provide a brief outline of it here. Using lab-reared lines of *Astyanax* descended from the eyed surface fish population of Balmorhea Springs State Park (Texas, USA) and the eyeless cavefish population of Cueva de El Pachón (Tamaulipas, Mexico), we mated a single surface fish female with a single cavefish male in order to generate several F_1_ hybrids. We then mated a single pair of these F_1_ hybrids in order to generate 384 F_2_ hybrid intercross progeny that displayed a range of eyed and eyeless phenotypes. Each hybrid was raised individually within a 500 mL tank and fed a diet of living *Artemia* larvae. We raised these F_2_ to either 1 or 2 years of age. Because of the limitations of tank size, all hybrids measured approximately the same length (∼3.0 cm), despite their difference in age. Following euthanasia, we fixed each fish in 4% PFA for 12 hours at room temperature, then rinsed the fish with 1× PBS and stored them in 100% MetOH at −80° C.

### Ethics

This study was approved by the University of Maryland College Park Institutional Animal Care and Use Committee (UMCP IACUC Protocol R-12-53 to WRJ). All fish were sacrificed using a lethal dose of 250 mg/L buffered MS-222 following our approved UMCP IACUC protocol.

### Histology and Retinal Measurements

We measured the thickness of each retinal layer from histological sections of 115 randomly chosen F_2_ individuals. We dissected the left eye from each fish, rinsed it in 1× PBS, and then re-fixed it in 4% PFA for 2 hours at room temperature. We dehydrated each eye in EtOH and Histo-clear^TM^ (National Diagnostics, Atlanta, GA, USA) using a series of washes (25%, 50%, 75%, and 3×100%) at room temperature for 5 and 20 minutes, respectively. Following dehydration, we perfused each eye with Paraplast® wax (Sigma-Aldrich, St. Louis, MO, USA) using another series of washes (25%, 50%, 75%, and 2×100%) at 57° C for 20 minutes, although we allowed the last wash to continue overnight. We then embedded each eye in a paraplast block and cut it into 20 µm sections using an American Optical 820 rotary microtome (Southbridge, MA, USA). We placed the sections in water on glass slides coated with poly-L-lysine and allowed them to flatten on a slide warmer overnight. Following sectioning, we de-waxed each slide in a bath of 100% histoclear and then stained the sections with Hematoxylin and 0.25% Eosine (Sigma-Aldrich) following standard protocols. We affixed a coverslip to each slide using Entellan® rapid mounting media (Electron Microscopy Sciences, Hatfield, PA, USA) and then imaged all sections on a Zeiss Axioskop2 confocal microscope equipped with a 40× Ph2 Plan-NEOFLUAR lens (Zeiss, Göttingen, Germany). Using a Zeiss AxioCam digital camera, we photographed images of each retinal section from the optic nerve to the ora serrata (Figure 1). Following imaging, we manually recorded the thickness of each retinal layer using the ‘Length’ tool in the program AxioVision 4.7 (Zeiss) and averaged the results for each individual. In addition to these retinal measurements, we also photographed the left lateral side of each fish and used AxioVision to measure the standard length (the length from the anterior to posterior of the fish, starting with the nose and ending at the caudal peduncle) as well as the total size (area, in µm^2^) of the left eye.

### RAD-seq Libraries and Genotyping

We identified genetic polymorphisms for QTL mapping using sequenced restriction-site associated DNA tags (RAD-seq) [28]. RAD-seq is a cost-effective method of genotyping that takes advantage of Illumina sequencing technology to simultaneously identify and genotype single nucleotide polymorphisms (SNPs) throughout the genome of non-model species. Our method for RAD-seq library construction and sequencing followed the protocols described in Etter et al. [28] and O'Quin et al. [29], with a few exceptions. Briefly, we used 10–20 mg of fin tissue to extract genomic DNA from 115 F_2_ hybrid individuals using a DNeasy Blood & Tissue extraction kit (Qiagen, Valencia, CA, USA). We quantified each sample using a Quant-iT^TM^ dsDNA High-sensitivity kit (Invitrogen, Grand Island, NY, USA) and a BioTek FLx800 fluorometer (Winooski, VT, USA), then digested 1 µg of DNA from each sample with the restriction enzyme *SbfI*-HF (New England Biolabs, Ipswich, MA, USA). We ligated one of 32 unique Illumina Solexa© P1 adaptors to each sample and then combined 3.75 µL (0.0625 µg) DNA from 32 individuals into a common library. We randomly sheared the DNA in each library to 500 bp using a Covaris S220 focused ultrasonicator and then size-selected fragments between 300–500 bp from a 2% agarose gel. We cleaned the fragments with a MinElute Gel Extraction kit (Qiagen), performed blunt-end repair with a Quick Blunting enzyme kit (New England Biolabs), and cleaned the products with a QiaQuick PCR Purification kit (Qiagen). We added adenine overhangs to the 3′ ends of each DNA library using a Klenow 3′–5′ exo kit (New England Biolabs) and cleaned the products with another QiaQuick PCR Purification kit. We then ligated a single Illumina Solexa© P2 adaptor to each DNA library and PCR amplified 15 µL of library for 16 cycles in a 100 µL reaction using modified amplification primers and Phusion Taq with HF Buffer. We purified the amplified libraries using a MinElute Gel Extraction kit eluted with 20 µL Buffer EB. Finally, we quantified each amplified library using an Agilent Bioanalyzer (Santa Clara, CA, USA) and then sequenced them single-end for 100 cycles on an Illumina HiSeq 1000 at the University of Maryland Institute for Bioscience & Biotechnology Research. Following sequencing, we filtered the raw sequence reads for quality (Q20 across 90% of the read) using the FASTX toolkit [30]. Finally, we used the function *process_radtags* within the program Stacks v0.998 [31] to further filter reads that did not contain complete *SbfI* and barcode sites, and to sort individuals within each library based on the sequence of their 5 bp barcode. We deposited this final set of sequences in the NCBI Sequence Read Archive (SRA) under BioProject Accession SRP017999.

We used the program Stacks v0.988 for the remainder of our genotyping analyses. We used the Stacks function *denovo_map*.*pl* to: (a) assemble the reads of each individual into unique loci (called ‘stacks’) and identify heterozygous alleles, (b) match orthologous stacks from the surface fish and cavefish P_0_ into a common catalog of loci and identify SNPs between them, and (c) match the stacks of all F_2_ against this parental catalog and infer genotypes at each locus. The parameters we used for sequence assembly allowed for a maximum of two mismatches when assembling the reads a single individual into a common stack, and one mismatch when matching the stacks from each individual against the parental catalog. The parameters we used for genotyping required the genotypes of all F_2_ to be supported by at least 20 sequencing reads, homozygous genotypes to have a maximum minor allele depth of 2%, and heterozygous genotypes to have a minimum minor allele depth of 8%. Genotypes with minor allele depths between 2–8% were considered ambiguous and called as ‘missing’. We found that these conservative genotyping criteria limit the inclusion of genotyping errors in downstream analyses. Following genotyping, we filtered the final dataset by excluding markers that: (a) were not differentially fixed between the surface fish and cavefish P_0_, (b) included missing genotypes in more than 25% of the F_2_, (c) had genotype frequencies that were outside of Hardy-Weinberg equilibrium at a Bonferroni-corrected threshold of α = 0.05, and (d) exhibited a maximum pair-wise recombination fraction greater than 90.9%. This later criterion is used to exclude alleles which may be “switched” (e.g., surface fish alleles called as cavefish alleles), indicating possible genotyping error in the P_0_ [32]. Finally, in addition to these RAD-seq loci, we also included 235 microsatellite and candidate gene markers that were indentified in previous studies [18], [26], [27].

### Linkage Mapping and QTL Scans

We performed genetic linkage and QTL analyses in the program R/qtl (Broman et al. 2003) following the protocols described in Broman [32] and Broman and Sen [33]. For genetic linkage mapping, we grouped the RAD-seq and microsatellite markers into linkage groups by specifying a maximum recombination distance of 0.35 and minimum LOD threshold of 6. We then ordered markers along each linkage group by iteratively rippling the order of eight markers at a time and choosing the order that required the fewest crossovers. We performed larger-scale changes in marker order manually and then estimated genetic distances using the Kosambi [34] map function. Finally, we calculated a LOD score for each genotype in order to identify statistically unlikely events such as double crossovers within a small region of the genetic map. We excluded these potentially erroneous genotypes from further analysis. Following linkage mapping, we scanned the genome for QTL associated with the thickness of individual retinal layers using *stepwiseqtl*, a model selection algorithm for multiple QTL mapping [33], [35]. We calculated the LOD of association between the thickness of each retinal layer and the genotypes at each marker (and markers simulated every 2.5 cM) using Haley-Knott regression, while including overall body size (standard length), eye size (area), and age at sampling (1 or 2 years old) as covariates. We assessed the statistical significance of the resulting LOD scores by calculating the 95^th^ percentile of genome-wide maximum penalized LOD scores for each retinal layer using 1000 random permutations of the genotypic and phenotypic data. We defined confidence intervals for the position of the final QTL using 95% Bayesian credible intervals expanded to the nearest genotyped marker.

### Comparative Mapping

To identify candidate genes within each QTL region, we anchored our *Astyanax* linkage map to the genome of the model species zebrafish (*Danio rerio*). To do this, we first downloaded the latest version of the zebrafish genome (Zv9/danRer7) from the University of California Santa Cruz (UCSC) Genome Browser Gateway (genome.ucsc.edu, accessed July 30 2012). We then built a searchable database of this genome and BLASTed the consensus sequences of each RAD-seq and microsatellite locus to this database using the discontinuous mega-blastn option of the program blast v2.2.25+ [36]. For RAD-seq loci, we retained the top hit with an expectation (E) value of 1 or lower; for microsatellite loci that had been anchored to the zebrafish genome in a previous analysis [26], we retained the top hit consistent with this previous result, no matter the E score. We used all anchored sites to identify regions of synteny between the zebrafish and *Astyanax* genomes which we visualized using the program Circos v0.62 [37]. We then downloaded a list of all zebrafish genes within our QTL regions using the UCSC RefSeq track. We searched for candidate genes within each anchored region by identifying those genes that are: (a) differentially expressed between *Astyanax* surface fish and cavefish, (b) implicated in the degeneration or function of specific retinal layers, or (c) important for eye development or maintenance.

## Results

### Retinal Thickness

We measured the thickness of individual retinal layers from the left eye of 115 randomly selected surface fish x cavefish F_2_ hybrids. Among these F_2_ hybrids, we observed a continuous range of retinal phenotypes between the two surface fish and cavefish extremes–from individuals that exhibited well-developed retinas with clearly defined layers, to those with poorly-developed retinas missing the RPE, PCL and ONL (Figure 1). In all, we measured retinal thickness across more than 1,000 retinal sections, ranging from 1–38 sections per individual (average: 11.26 sections per individual ±6.91 standard deviations). In order to account for intra-retinal variation caused by changes in location or oblique sectioning, we computed the average thickness of each retinal layer from all sections of the same F_2_ hybrid. Standard deviations in the thickness of all layers from each individual's retinal sections were generally small and ranged from 0.01–31.86 µm with a mean of 3.79 µm.

Total retinal thickness averaged for each individual ranged from 54.22–197.90 µm (average: 120.46±28.50 s.d.). The thickest layers were the RPE and PCL (35.54±13.04 µm and 21.32±9.11 µm, respectively). These two layers also exhibited the greatest variation in thickness and were even missing from a few individuals (Figure 1). However, some of this variation may be due to measurement error since these layers frequently overlapped each other in the retinal sections. Additionally, several individuals exhibited RPE that were albino or hypopigmented, although these did not necessarily correspond to individuals with the thinnest retinas (Figure 1). The next thickest layers were the IPL and INL (16.86±5.01 µm and 20.31±5.81 µm), which were also the second-most variable. These layers were followed by the OPL and ONL (8.46±2.84 µm and 12.59±4.65 µm). The thinnest layer was the GCL (5.38±1.59 µm), which is consistent with this layer being generally no thicker than a single ganglion cell. However we did notice considerable variation in the development of the GCL. In particular, several individuals exhibited large gaps between ganglion cells, which typically resulted in thinner GCL values when averaged across multiple sections. Thus, although the relative size of a ganglion cell may not differ among the hybrid F_2_, variation in ganglion cell development could still be captured with our measurements. Although we measured the thickness of each layer individually, correlation analysis reveals that most layers were positively correlated with one another, especially those that are directly adjacent (Table 1). Our results reveal dramatic variation in the thickness and development of the retina of *Astyanax* F_2_ hybrids. However, we note that all of the F_2_ we sampled had eyes and retina, the smallest of which are representative of the most well-developed eyes in cavefish. This lack of diversity at the cavefish-end of the phenotypic spectrum, as well as possible measurement error in the RPE and PCL, might limit our power to detect quantitative trait loci (QTL) for all retinal layers.

**Table 1:** Pearson's correlation coefficients for retinal thickness among 115 *Astyanax* F_2_ hybrids.

Layer	IPL	INL	OPL	ONL	PCL	RPE
GCL	0.47^****^	0.69^****^	0.69^****^	0.55^****^	0.17^n.s.^	−0.05^n.s.^
IPL		0.71^****^	0.47^****^	0.35^**^	0.20^n.s.^	0.02^n.s.^
INL			0.63^****^	0.53^****^	0.24^n.s.^	−0.09^n.s.^
OPL				0.65^****^	0.47^****^	0.14^n.s.^
ONL					0.40^***^	0.21^n.s.^
PCL						0.70^****^

Bonferroni-corrected P-value < ^*^0.05, ^**^0.01, ^***^0.001, ^****^0.0001; n.s.: not significant.

GCL: ganglion cell layer; IPL: inner plexiform layer; INL: inner nuclear layer; OPL: outer plexiform layer; ONL: outer nuclear layer; PCL: photoreceptor cell layer; RPE: retinal pigment epithelium.

### RAD-seq Genotyping

Illumina sequencing of the reduced-representation RAD-seq libraries yielded ∼4.6 million reads per individual. These reads assembled into ∼79,000 unique loci in which we identified an average of 10,000 SNPs per individual (Table S1). After filtering these SNPs based on their fixation in the surface fish and cavefish parents, percentage of missing genotypes, Hardy-Weinberg equilibrium frequency, and pairwise recombination fraction, we retained a conservative set of 463 RAD-seq SNPs and 235 microsatellite markers. The total fraction of missing genotypes across the entire set of 698 markers and 115 individuals is less than 10%, while the frequencies of each genotypic class approximate Hardy-Weinberg proportions (25.9% homozygous for surface fish alleles, 22.8% homozygous for cavefish alleles, and 51.2% heterozygous).

### Linkage Mapping and Anchoring to the Zebrafish Genome

**Figure 2:**
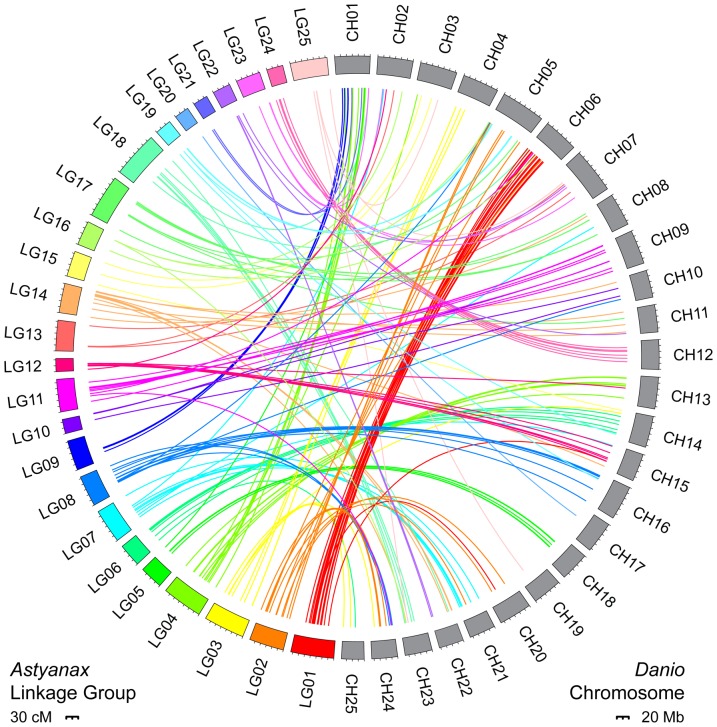
Comparative mapping of the *Astyanax* and zebrafish genomes. Colored boxes represent *Astyanax* linkage groups 1−25; grey boxes represent zebrafish (*Danio rerio*) chromosomes 1–25. Colored lines link genetic markers from *Astyanax* to their BLAST position in zebrafish. Circle plot created with Circos [37].

Linkage mapping assembled the 698 RAD-seq, microsatellite, and candidate gene markers into 25 distinct linkage groups spanning a total of 1835.5 cM (Figure S1; Table S2). The average intermarker distance of this linkage map is 3.1 cM. Since *Astyanax* have a karyotype of 25 haploid chromosomes spanning 1.2 Gb and ∼1730 cM [18], [26], [38], this linkage map represents the densest and most complete genetic map of the *Astyanax* genome to date. As in a previous anchoring study [26], we found widespread synteny between the *Astyanax* and zebrafish (*Danio rerio*) genomes (Figure 2; Table S2). Our BLAST analysis anchored a total of 173 *Astyanax* markers to the zebrafish genome, including 68 RAD-seq loci, 81 previously-mapped microsatellites, and 24 candidate genes. Of the 81 previously-mapped loci, 12 had E-values >1.0 and 21 had no hits at all. However, using the positions of surrounding markers, we were able to estimate positions for these markers based on their location in Gross et al. [26]. Every *Astyanax* linkage group and zebrafish chromosome included at least one anchored marker (Figure 2; Table S2). This newly anchored map will facilitate the identification of candidate gene regions following QTL mapping.

### QTL Mapping and Candidate Gene Identification

**Figure 3:**
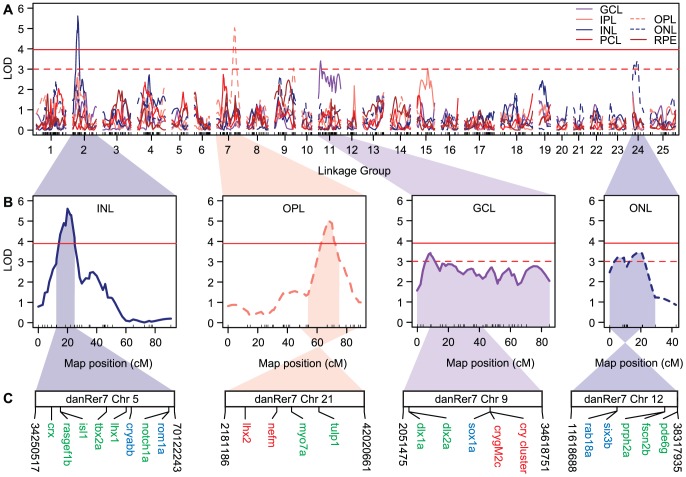
QTL mapping of retinal thickness in F_2_ hybrids. (A) LOD scores of association between genotypes on each linkage group and the thickness of seven retinal layers. Retinal layers include the ganglion cell layer (GCL), inner plexiform layer (IPL), inner nuclear layer (INL), outer plexiform layer (OPL), outer nuclear layer (ONL), photoreceptor cell layer (PCL), and retinal pigment epithelium (RPE). Horizontal lines represent the genome-wide significant threshold at *P* = 0.05 (solid) and 0.15 (dotted). (B) Significant QTL include those for INL thickness on *Astyanax* LG 2, OPL thickness on LG 7, GCL thickness on LG 11, and ONL thickness on LG 24. Highlighted regions represent the 95% Baysian credible interval for each QTL position. (C) Markers within and around each QTL region map to four ∼35 Mb regions of the zebrafish (*Danio rerio*) genome on chromosomes 5, 21, 9, and 12. Within each region, we find several candidate genes that are either: (red) differentially expressed between *Astyanax* surface fish and cavefish; (green) implicated in vertebrate retinal degeneration; or (blue) important for eye development and function (see Discussion).

Following linkage analysis, we scanned the *Astyanax* genome for QTL associated with the thickness of each retinal layer while controlling for eye size, standard length, and age at sampling. Our multiple QTL mapping approach identified 2 QTL at the genome-wide significance threshold of *P* = 0.05 (LOD = 3.96) and 2 additional QTL at the more liberal cutoff of *P* = 0.15 (LOD = 3.00) (Figure 3). Because retinal degeneration in *Astyanax* is a complex trait governed by multiple loci [7], our current analysis of 115 F_2_ is probably underpowered to detect all possible QTL. Therefore, we chose to use the more liberal threshold of P = 0.15 to identify additional loci, although we note that these latter QTL will need to be confirmed in future analyses.

**Figure 4:**
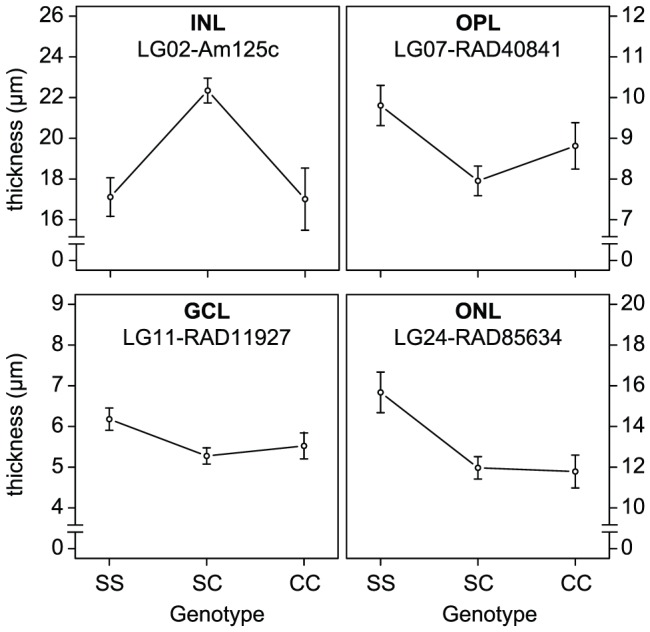
Mean thickness of retinal layers among genotypic classes at each QTL. Mean values of inner nuclear (INL), outer plexiform (OPL), ganglion cell (GCL), and outer nuclear (ONL) layer thickness are plotted for genotypic classes at the peak marker for each QTL (see Figure 3). The distributions of mean OPL, GCL, and ONL thickness indicate that cavefish alleles are inherited dominantly and contribute to a clear decrease in retinal thickness. The distribution of mean INL thickness indicates that cavefish alleles for this QTL may be inherited overdominantly. Error bars indicate standard deviation. SS: surface fish homozygote; CC: cavefish homozygote; SC: heterozygote.

The two strongest QTL for retinal degeneration map to linkage groups (LG) 2 and 7 and correspond to variation in INL and OPL thickness (Figure 3). The INL QTL on LG 2 has a maximum LOD of 5.62 and spans a region of 12.3 cM. It explains 10% of the variation in INL thickness and cavefish alleles at this locus contribute to a 4.47 µm change in INL size (Table 2). Interestingly, however, individuals with heterozygous genotypes at this QTL have thicker INL than either the surface fish or cavefish homozygotes, indicating overdominance (Figure 4). The OPL QTL on LG 7 has a maximum LOD of 5.04 and spans a region of 17.2 cM (Table 2). It explains 11.15% of the variation in OPL thickness and cavefish alleles at this locus contribute to a clear 0.81 µm reduction in OPL thickness, with cavefish alleles dominant to those from surface fish (Table 2; Figure 4).

**Table 2:** Summary statistics of four QTL for *Astyanax* retinal degeneration.

Retinal	LG	Position	LOD	P-value	PVE	Peak	BCI	Dom.	Dom.	Add.
Layer		(cM)			(%)	Marker	(cM)	Dev.	Effect	Effect
INL	2	20.00	5.62	0.002	10.00	Am125c	12.5–24.8	5.28	4.47	−1.29
OPL	7	67.50	5.04	0.003	11.15	RAD_40841	54.2–71.4	−1.31	−0.81	−1.31
GCL	11	8.62	3.41	0.157	6.53	RAD_11927	3.7–85.3	−0.55	−0.50	−0.44
ONL	24	20.00	3.42	0.145	9.17	RAD_85634	0.0–29.3	−1.79	−2.04	−2.03

LG: linkage group; LOD: logarithm of the odds; PVE: percent variance explained; BCI: Bayesian credible interval; Dom. Dev.: dominance deviation; INL: inner nuclear layer; OPL: outer plexiform layer; GCL: ganglion cell layer; ONL: outer nuclear layer.

The two weaker QTL are found on LG 11 and 24 and correspond to GCL and ONL thickness (Figure 3). In addition to lower LOD scores, these weaker QTL generally explain a smaller proportion of the variance for each layer and have credible intervals that span a larger portion of each LG. The GCL QTL on LG 11 has a maximum LOD of 3.41, although its LOD is elevated across the entire chromosome; consequently, the credible interval for this QTL covers nearly the entire length of LG 11 (Table 2; Figure 3). The peak marker for this QTL explains 6.53% of the variance in the GCL development, and cavefish alleles contribute to a 0.50 µm reduction in GCL thickness (Table 2). This QTL could represent differences in the size of individual GCL cells, but more likely represents variation in the development of the GCL, where less developed layers exhibit more gaps between cells. The ONL QTL on LG 24 has a maximum LOD of 3.42 and spans a region of 29.3 cM. It explains 9.17% of the variance in ONL thickness and cavefish alleles result in a 2.04 µm reduction in ONL size (Table 2). Cavefish alleles at both QTL are dominant to those from surface fish (Figure 4). We provide a list of all retinal measurements and marker genotypes used to identify these QTL in Table S3.

The QTL intervals on *Astyanax* LGs 2, 7, 11, and 24 each map to distinct regions of the zebrafish genome on chromosomes 5, 21, 9, and 12 (Figure 3; Table S2). On average, each candidate QTL region spans 35 Mb of the zebrafish genome. These regions contain numerous candidate genes that could contribute to retinal degeneration in *Astyanax*. For example, several genes are differentially expressed between surface fish and cavefish (shown in red on Figure 3) [11], [39]. Other genes in these regions have been shown to influence retinal degeneration or to affect GCL, INL, OPL, or ONL function (shown in green on Figure 3) (e.g., [3]). The remaining candidates play an important role in determining eye and lens size (shown in blue on Figure 3). The role of these potential candidates is discussed in more detail below.

## Discussion

### Astyanax Retinal Degeneration

Most work in *Astyanax* has focused on the evolutionary implications of eye loss, and none have investigated the genetic basis of retinal degeneration specifically, especially in the context of human ocular disease. Our cross of *Astyanax* surface fish and cavefish produced offspring that exhibit dramatic variation in the overall size and development of their retinas. Specifically, we found considerable variation in the thickness of the INL, ONL, PCL, and RPE, and variation in the development of the GCL (Figure 1). The observation that nearly all of these progeny retain the full complement of retinal layers, including photoreceptors, suggests that most individuals completed the earliest stages of retinal development [11]; therefore, it is likely that the variation we observe in the thickness of individual retinal layers is the result of degeneration combined with other intrinsic factors. The range of retinal phenotypes we observe is consistent with previous analyses of *Astyanax* retinal ultrastructure, although it is somewhat limited at the cavefish-end [7], [8]. An additional caveat is that the wide variation in PCL and RPE thickness could have limited our ability to detect quantitative trait loci (QTL) for these two layers. Most importantly, however, our results suggest that the PCL, ONL, INL, and GCL are the layers most impacted by apoptosis in cavefish–the same retinal layers impacted in human cases of retinitis pigmentosa and albinism [3], [25]. These results suggest that future work should focus on the mechanisms responsible for degeneration in these specific layers. However, it is also possible that variation in the thickness of one layer actually reflects variation in multiple cell types, especially since most layers were positively correlated (Table 1) and apoptosis is observed throughout the cavefish retina [11], [12].

### Genetic mapping and comparison to previous QTL for eye size

Previous genetic maps of the *Astyanax* genome used to detect QTL for eye size and other degenerative traits had more linkage groups than expected chromosomes, and anchoring with these maps failed to detect synteny with all zebrafish chromosomes [18], [26], [40]. In this analysis, we constructed a high-density map of the *Astyanax* genome using >400 new genetic markers derived from sequenced restriction-site associated DNA (RAD-seq) tags and >200 markers used in previous *Astyanax* maps. With nearly 700 genetic markers, this new genetic map represents the densest and most complete map of the *Astyanax* genome to-date. Linkage mapping clearly resolved these markers into 25 linkage groups (LG), matching the number of expected haploid chromosomes [38]. Additionally, with this map we are able to anchor each *Astyanax* linkage group to the zebrafish genome with one or more markers. For example, although Gross et al. [26] failed to detect any synteny between the *Astyanax* genome and zebrafish chromosome 11, our additional RAD-seq loci anchored zebrafish chromosome 11 to *Astyanax* LG 14 and 17 (Figure 2; Table S2). This dense genetic map, which includes both new and previously identified genetic markers, grants us unparalleled resolution to compare the location of previous eye QTL to the retinal QTL found in this study, and to identify candidate genes.

Eye loss in *Astyanax* is a complex trait controlled by numerous genetic factors. In addition to starting with a smaller eye field, *Astyanax* cavefish also undergo lens and retinal apoptosis. Consequently, previous studies of *Astyanax* eye and lens size have identified as many as 12 QTL for these traits [18], [26], [40]. Several eye and lens size QTL overlap, suggesting that the same or tightly-linked genetic factors may control both traits. This hypothesis has been partially confirmed by experimental studies that demonstrate that lens transplants restore eye development in *Astyanax* cavefish [13] and promote retinal cell survival [22]. These results suggest that the genes and genetic pathways controlling lens and eye degeneration should also control retinal degeneration. But until now, the linkage between eye, lens, and retinal QTL has remained unclear.

We identified four QTL controlling retinal INL, OPL, GCL, and ONL thickness on linkage groups 2, 7, 11, and 24 (Figure 3). Consistent with the observation that eye size in *Astyanax* is a complex genetic trait controlled by many alleles of small effect [8], [17], [18], we found that most of these QTL explain less than 10% of the variance in any affected layer (Table 2). Furthermore, we also found that cavefish alleles at these loci are dominant or overdominant to those from surface fish, which is also consistent with QTL analyses of *Astyanax* eye size [18]. Significantly, the two strongest QTL for INL and OPL thickness on LG 2 and 7 contain or are tightly linked to markers associated with eye size in previous analyses (see Table S2 as well as LG 4 and 6–8 in [18]). The QTL for INL thickness on LG 2 is also associated with lens size [18]. These results suggest that lens and retinal degeneration could influence *Astyanax* eye size either directly through the action of shared developmental genes or indirectly through a combination of factors such as pleiotropy and genetic hitchhiking. Interestingly, eye size, lens degeneration, and defective retinal morphology are frequently correlated in humans with anopthalmia/microphthalmia caused by mutations in *sox2* and *vsx2/chx10* [15].

### Candidate genes for retinal degeneration and eye size

We anchored our *Astyanax* genetic map to the zebrafish genome in order to identify candidate genes within our retinal QTL. However, even with our new genetic map, the QTL we identify still correspond to very large (∼35 Mb) regions of the zebrafish genome and contain numerous candidate genes (Figure 3). But using the fact that each QTL corresponds to changes in a specific retinal layer, we narrowed the pool of possible candidates by discarding those unrelated to GCL, INL, OPL, or ONL function. Although the genes we describe below do not represent an exhaustive list of possible loci, we believe they represent the best candidates for retinal degeneration in *Astyanax* given their association with these retinal layers and ocular diseases such as retinitis pigmentosa and anophthalmia/microphthalmia.

The QTL on LG 2 corresponds to INL thickness and maps to zebrafish chromosome 5 (Figure 3). This genomic region is also associated with eye size and lens reduction in *Astyanax* [18]. Although none of the genes in this region are known to be differentially regulated between *Astyanax* surface fish and cavefish, several have been shown to influence INL thickness, eye growth, and lens function. The most obvious candidates for INL thickness include *cone-rod homeobox* (*crx*), *ISL LIM homeobox 1* (*isl1*), *rasgef domain member 1b* (*rasgef1b*), and *LIM homeobox 1* (*lhx1*). All four genes are associated with the differentiation or localization of amacrine, bipolar, or horizontal cells that make up the INL [41], [42], [43]. The gene *notch1a* is also located within this region. Although not associated with INL thickness, mutations within *notch1a* are associated with a more general apoptosis in retina progenitor cells [44]. In addition to the genes for INL function, two other genes within this region may play a role in eye size, including *retinal outer segment membrane protein 1a* (*rom1a*) and *T-box 2a* (*tbx2a*). Like *crx*, which is important for INL and photoreceptor differentiation, *rom1a* is also necessary for photoreceptor differentiation, and this gene has already been highlighted as a candidate for eyeloss in *Astyanax* [26]. Therefore, mutations affecting either *crx* or *rom1a* could theoretically explain the reduced eye size and slow rate of photoreceptor differentiation found in *Astyanax* cavefish. Similarly, the gene *tbx2a* is necessary for eyefield specification, and *tbx2*-kockout mice have smaller eye cups, increased retinal apoptosis, and abnormal GCL projections [45]. The INL candidate gene *isl1* could also be associated with eye size since it is co-expressed with *shh* in mice [42]. Since *shh* expression is expanded in *Astyanax* cavefish and *shh* mRNA leads to eye reduction when injected into developing surface fish [20], it is possible that mutations within *isl1* could simultaneously contribute to both retinal degeneration and eye reduction. Finally, the gene *cryabb* is also found within this region and is highly expressed within the zebrafish lens [46]. This may serve as a candidate for the lens size QTL found in this region [18].

The QTL on LG 7 corresponds to OPL thickness and maps to zebrafish chromosome 21 (Figure 3). In addition to OPL thickness, this genomic region is also associated with eye size in *Astyanax* [18]. Two genes within this area are differentially expressed between *Astyanax* surface fish and cavefish [11], [39]. The first gene, *LIM homeobox 2* (*lhx2*), is expressed in the *Astyanax* INL and cilliary marginal zone where retinal progenitor cells are made [11]. It is one of the few genes expressed earlier in cavefish than surface fish, and it is also expressed in cavefish for a longer period of time [11]. In mice, *lhx2*-knockouts are eyeless [47], while zebrafish *lhx2* mutants retain small eyes with disorganized retinas and few amacrine cells [48]. The second candidate gene, *neurofilament medium polypeptide a* (*nefma*), is downregulated in cavefish [39]. In mice, *nefma* is expressed within the GCL and possibly the INL as well [49]. Since both *lhx2* and *nefma* affect cells in the INL, which is directly adjacent to and highly correlated with the OPL (Figure 1; Table 1), they represent excellent candidates for the control of eye size and OPL thickness in cavefish. Two other genes within this region are also directly associated with OPL function. In mice, *myosin 7a* (*myo7a*) is associated with synaptic trafficking. It is expressed in the OPL, and mutations within this gene lead to changes in ONL, OPL, and INL thickness [50]. Interestingly, mutations within *myo7a* are also associated with defective melanosome distribution and photoreceptor phagocytosis in the RPE [51], [52]. Thus, it is possible that mutations affecting *myo7a* could contribute to both OPL thickness and RPE hypopigmentation in cavefish. The second gene, *tubby-like protein 1* (*tulp1*), is also expressed within the OPL and is necessary for normal synaptic development between the ONL and INL [53], [54]. Like *myo7a*, mutations within *tulp1* are also associated with photoreceptor degeneration and retinitis pigmentosa [54].

The QTL on LG 11 corresponds to GCL development and maps to zebrafish chromosome 9 (Figure 3). Two genes within this region are differentially regulated between *Astyanax* surface fish and cavefish [39]. The first gene is *gamma M2c crystallin* (*crygm2c*) and the second gene maps to an unnamed protein in the middle of a cluster of crystallin paralogs. Both genes are downregulated in the cavefish lens (along with six other crystallin genes), which undergoes apoptosis even earlier than the cavefish retina [11]. It is unlikely that *crygM2c* or the other *crystallins* directly affect retinal degeneration or GCL development, but it is possible that they influence retinal degeneration indirectly through the inductive role of the lens [13], [22], [23]. Another lens candidate gene, *sry-box containing gene 1a* (*sox1a*) also occurs in this region and is expressed in the zebrafish lens [55]. Since *sox1a* is required for gamma crystallin expression [55], this gene is yet another candidate for lens apoptosis. Apart from these crystallin genes, the most likely candidates for direct control of GCL thickness are two *distal-less homeobox* (*dlx*) paralogs, *dlx1a* and *dlx2a*. In mice, both *dlx1* and *dlx2* are expressed within the GCL and INL, and both genes play a role in the development and survival of retinal ganglion cells [56], [57]. Thus, mutations affecting either or both *dlx* paralogs could conceivably reduce GCL thickness in *Astyanax* cavefish.

Finally, the QTL on LG 24 corresponds to ONL thickness and maps to zebrafish chromosome 12 (Figure 3). None of the genes within this region have been shown to be differentially regulated between *Astyanax* surface fish and cavefish, although three genes are associated with photoreceptor degeneration and retinitis pigmentosa. The first gene is *peripherin 2a* (*prhp2a/rds*) [58]. Like *rom1a* on zebrafish chromosome 5 (Figure 3), *prph2a* is classically associated with photoreceptor differentiation and degeneration in mice. *Prph2a* is expressed within rod outer segments where it functions in photoreceptor disk morphogenesis. In mice, mutations within *prph2a* result in photoreceptor apoptosis [58]. The second gene, *fascin 2b* (*fascn2b*), is expressed within the inner segment of photoreceptors where it cross-links actin filaments and may function in *rhodopsin* trafficking [59]. Like *prph2*, mutations within *fascin 2b* lead to photoreceptor death. The last gene, *rod phospodiesterase 6g* (*pde6g*), functions in the regulation of cyclic GMP in rods following exposure to light, and mutations within this gene are associated with retinitis pigmentosa [60], [61]. All three candidates are especially interesting in light of the fact that *rhodopsin* is differentially expressed between *Astyanax* surface fish and cavefish [39]. Assuming that photoreceptor cell death will lead to a decrease in the number of photoreceptor nuclei within the ONL, mutations affecting any or all of these genes could explain the decrease in ONL thickness found in *Astyanax* cavefish (Figure 4). Finally, in addition to these photoreceptor-specific genes, two genes within this QTL are associated with micropthalmia and more general developmental delays. *Ras-associated protein 18a* (*rab18a*) causes Warburg micro syndrome in humans and mutations within this gene cause microphthalmia, while mutations in a close paralog, *rab18b*, also cause delayed retinal development and abnormal retinal lamination [62]. A second gene, *sine oculis homeobox 3b* (*six3b/six6*), regulates retinal neurogenesis and is also associated with anophthalmia/micropthalmia [63].

Future work will fine-map the causative mutations within these QTL through a combination of increased marker density, greater sampling within inbred and outbred populations, candidate gene analyses, and the addition of more high-throughput phenotyping methods such as optical coherence tomography.

### Conclusions

We have performed a quantitative genetic analysis of retinal degeneration in the characin fish *Astyanax mexicanus*. We identify at least four QTL associated with degeneration in specific layers of the retina, and identify numerous candidate genes specific to these retinal layers. Our results suggest that mutations affecting retinal degeneration in *Astyanax* may also influence the evolution of eye size in this species. Finally, this work also demonstrates that the eyed surface- and eyeless cave-dwelling forms of *Astyanax mexicanus* can serve as useful evolutionary mutant models for human ocular disease.

## Supporting Information

Figure S1 **Linkage map of the** ***Astyanax*** **genome.** Linkage map constructed from 115 F_2_ hybrid progeny of a cross between a single surface fish female (Texas) and a single cavefish male (Pachón). The map includes 698 markers assembled into 25 linkage groups that collectively span 1835.5 cM. Markers names identified in black denote RAD-seq markers developed in this study; those in blue denote markers developed in previous studies (see Materials and Methods).
(EPS)

Table S1 **RAD-seq assembly statistics for all F_2_ hybrids.**
(XLSX)

Table S2 **Linkage and comparative mapping position of all genetic markers used in this study, including their LOD of association with retinal thickness.**
(XLSX)

Table S3 **Retinal measurements and marker genotypes used to scan the** ***Astyanax*** **genome for QTL related to retinal degeneration.**
(CSV)
